# Supplementary material for: cOmicsArt—a customizable Omics Analysis and reporting tool
Source: Bioinform Adv. 2025 Apr 1;5(1):vbaf067. doi: 10.1093/bioadv/vbaf067 (PMC12085238; doi:10.1093/bioadv/vbaf067)
Supplement: vbaf067_Supplementary_Data [file vbaf067_supplementary_data.zip › SupplementaryE_ShowcaseA_report.html]

Report


# ShinyOmics Report (25/07/2024)

This is a report generated by the
cOmicsART
application under version v0.1.0. Documentation on the user interface
can be found
here.

## Data Selection

### Info

**DataInput** - Uploaded Omic Type: Transcriptomics

The following data was used: DataMatrix.csv SampleAnno.csv entities
annotation\_showcase.csv

**DataInput** - The raw data dimensions are: 47643,
10

**DataInput** - Gene Annotation (SYMBOL and gene type)
was added

**DataInput** - chosen Organism: Mouse genes
(GRCm39)

**DataSelection** - The following selection was
conducted:

**DataSelection** - Samples: DataSelection - based on:
Organism: all

**DataSelection** - Entities: DataSelection - based on:
Ensembl\_ID: all

### Publication Snippet

The data was uploaded to cOmicsART (v. v0.1.0) a webapp to perform
explorative and statistical analysis with seamless integration to R
(Seep et. al. 2024). The webapp is majorly built with the shiny package
(v. 1.8.1.1) (Chang W, Cheng J, Allaire J, Sievert C, Schloerke B, Xie
Y, Allen J, McPherson J, Dipert A, Borges B (2024).\_shiny: Web
Application Framework for R\_. R package version 1.8.1.1,https://CRAN.R-project.org/package=shiny.). It is
currently running on R (v. 4.2.0) (R Core Team (2022). *R: A Language
and Environment for Statistical Computing*. R Foundation for
StatisticalComputing, Vienna, Austria. https://www.R-project.org/.). Unless otherwise stated,
all visulaizations were created using the ggplot2 package (v. 3.5.1)
(Wickham H (2016). *ggplot2: Elegant Graphics for Data Analysis*.
Springer-Verlag New York. ISBN978-3-319-24277-4, https://ggplot2.tidyverse.org.). The Transcriptomics
data was uploaded with the original dimensions of 47643 features and 10
samples. Gene annotation was added using the Mouse genes (GRCm39)mart
from Ensembl implemented within the biomaRt package (v. 2.54.1) (Durinck
S, Spellman P, Birney E, Huber W (2009). “Mapping identifiers for the
integration of genomic datasetswith the R/Bioconductor package biomaRt.”
*Nature Protocols*, *4*, 1184-1191.Durinck S, Moreau Y,
Kasprzyk A, Davis S, De Moor B, Brazma A, Huber W (2005). “BioMart and
Bioconductor: apowerful link between biological databases and microarray
data analysis.” *Bioinformatics*, *21*, 3439-3440.). No
sample selection was performed. No entitie selection was performed.

## Pre Processing

### Info

**PreProcessing** - Alaways done: removal of all
entities which are constant over all samples

**PreProcessing** - Preprocessing procedure -standard
(depending only on omics-type): Remove anything which row Count <=
10

**PreProcessing** - Preprocessing procedure -specific
(user-chosen): vst\_DESeq~Treatment

**PreProcessing** - The resulting dimensions are: 16125,
10

### Publication Snippet

For the transcriptomics data, DESeq2 was used for normalization and
VST transformation applied for visualisation of the normalized data (not
for statistical testing)(v. 1.38.3) (Love MI, Huber W, Anders S (2014).
“Moderated estimation of fold change and dispersion for RNA-seq data
withDESeq2.” *Genome Biology*, *15*, 550. doi:10.1186/s13059-014-0550-8https://doi.org/10.1186/s13059-014-0550-8.). The formula
for analysis was ~~ Treatment~ Treatment. The resulting dataset had
16125 features and 10 samples.

## Sample correlation

### Info

**SampleCorrelation** - The correlation method used was:
pearson

**SampleCorrelation** - The heatmap samples were colored
after Treatment

**SampleCorrelation** -

### Publication Snippet

The correlation between samples was calculated using the pearson
method. The resulting correlation matrix was visualized using the
pheatmap package(v. 1.0.12) (Kolde R (2019). *pheatmap: Pretty
Heatmaps*. R package version 1.0.12,https://CRAN.R-project.org/package=pheatmap.). The
correlation matrix was clustered with the complete linkage method using
correlation distance.

## PCA

### Info

**PCA** - The PCA was computed on the entire
dataset.

**PCA** - The following PCA-plot is colored after:
Treatment

**PCA** -

### Publication Snippet

Principal component analysis (PCA) was performed on the centered and
scaled data, implemented within the stats package (v.4.2.0) (R Core Team
(2022). *R: A Language and Environment for Statistical
Computing*. R Foundation for StatisticalComputing, Vienna, Austria.
https://www.R-project.org/.).

## PCA ScreePlot

### Info

**ScreePlot** - The scree Plot shows the Variance
explained per Principle Component

**ScreePlot** -

### Publication Snippet

The scree plot was generated to visualize the proportion of variance
explained by each principal component.

## PCA Loadings

### Info

**LoadingsPCA** - Loadings plot for Principle Component:
PC1

**LoadingsPCA** - Showing the the highest 10 and the
lowest 10 Loadings

**LoadingsPCA** - The corresponding Loadingsplot -

### Publication Snippet

The top 10 positive loadings and the top 10 negative loadings were
seleceted to assess an entities’ impact on the principal components

## PCA Loadings Matrix

### Info

**PCALoadingsMatrix** - Loadings plot for Principle
Components 1 till PC1

**PCALoadingsMatrix** - Showing all entities which have
an absolute Loadings value of at least0.05

**PCALoadingsMatrix** - The corresponding Loadings
Matrix plot -

### Publication Snippet

The loadings matrix was created by taking all absolute loading values
higher than 0.05 into account for the first 1The resulting matrix allows
a visual assessment of the impact of each entity accross multiple
principal components.

## Single Entitie

### Info

**Single Entitie** - The following single entitie was
plotted:

**Single Entitie** - Values shown are: data input

**Single Entitie** - Values are grouped for all levels
within: ()

**Single Entitie** - Test for differences:

**Single Entitie** - pairwise tested

**Single Entitie** -

### Publication Snippet

The expression of, Ppbp, was plotted. The values shown represent the
preprocessed data. If the a group of entities is selected through their
shared annotation, the median value is used as representative for those
entities for the respectice sampleValues are grouped for all levels
within the condition: Treatment). A test for differences was performed
using the t.test method. Pairwise tests were performed. The dotted line
represents the global mean. Boxplots are only shown if there are more
than 3 samples per group. The plot was extended to include and visualize
the statistical results with the R packge ggpubr(v. 0.6.0) (Kassambara A
(2023). *ggpubr: ‘ggplot2’ Based Publication Ready Plots*. R
package version 0.6.0,https://CRAN.R-project.org/package=ggpubr.).

## Single Entitie

### Info

**Single Entitie** - The following single entitie was
plotted:

**Single Entitie** - Values shown are: data input

**Single Entitie** - Values are grouped for all levels
within: ()

**Single Entitie** - Test for differences:

**Single Entitie** - pairwise tested

**Single Entitie** -

### Publication Snippet

The expression of, Osm, was plotted. The values shown represent the
preprocessed data. If the a group of entities is selected through their
shared annotation, the median value is used as representative for those
entities for the respectice sampleValues are grouped for all levels
within the condition: Treatment). A test for differences was performed
using the t.test method. Pairwise tests were performed. The dotted line
represents the global mean. Boxplots are only shown if there are more
than 3 samples per group. The plot was extended to include and visualize
the statistical results with the R packge ggpubr(v. 0.6.0) (Kassambara A
(2023). *ggpubr: ‘ggplot2’ Based Publication Ready Plots*. R
package version 0.6.0,https://CRAN.R-project.org/package=ggpubr.).

## Single Entitie

### Info

**Single Entitie** - The following single entitie was
plotted:

**Single Entitie** - Values shown are: data input

**Single Entitie** - Values are grouped for all levels
within: ()

**Single Entitie** - Test for differences:

**Single Entitie** - pairwise tested

**Single Entitie** -

### Publication Snippet

The expression of, Fos, was plotted. The values shown represent the
preprocessed data. If the a group of entities is selected through their
shared annotation, the median value is used as representative for those
entities for the respectice sampleValues are grouped for all levels
within the condition: Treatment). A test for differences was performed
using the t.test method. Pairwise tests were performed. The dotted line
represents the global mean. Boxplots are only shown if there are more
than 3 samples per group. The plot was extended to include and visualize
the statistical results with the R packge ggpubr(v. 0.6.0) (Kassambara A
(2023). *ggpubr: ‘ggplot2’ Based Publication Ready Plots*. R
package version 0.6.0,https://CRAN.R-project.org/package=ggpubr.).

## Single Entitie

### Info

**Single Entitie** - The following single entitie was
plotted:

**Single Entitie** - Values shown are: data input

**Single Entitie** - Values are grouped for all levels
within: ()

**Single Entitie** - Test for differences:

**Single Entitie** - pairwise tested

**Single Entitie** -

### Publication Snippet

The expression of, Dusp1, was plotted. The values shown represent the
preprocessed data. If the a group of entities is selected through their
shared annotation, the median value is used as representative for those
entities for the respectice sampleValues are grouped for all levels
within the condition: Treatment). A test for differences was performed
using the t.test method. Pairwise tests were performed. The dotted line
represents the global mean. Boxplots are only shown if there are more
than 3 samples per group. The plot was extended to include and visualize
the statistical results with the R packge ggpubr(v. 0.6.0) (Kassambara A
(2023). *ggpubr: ‘ggplot2’ Based Publication Ready Plots*. R
package version 0.6.0,https://CRAN.R-project.org/package=ggpubr.).

## Single Entitie

### Info

**Single Entitie** - The following single entitie was
plotted:

**Single Entitie** - Values shown are: data input

**Single Entitie** - Values are grouped for all levels
within: ()

**Single Entitie** - Test for differences:

**Single Entitie** - pairwise tested

**Single Entitie** -

### Publication Snippet

The expression of, Ppbp, was plotted. The values shown represent the
preprocessed data. If the a group of entities is selected through their
shared annotation, the median value is used as representative for those
entities for the respectice sampleValues are grouped for all levels
within the condition: Stimulation\_Treatment). A test for differences was
performed using the t.test method. Pairwise tests were performed. The
dotted line represents the global mean. Boxplots are only shown if there
are more than 3 samples per group. The plot was extended to include and
visualize the statistical results with the R packge ggpubr(v. 0.6.0)
(Kassambara A (2023). *ggpubr: ‘ggplot2’ Based Publication Ready
Plots*. R package version 0.6.0,https://CRAN.R-project.org/package=ggpubr.).

## Significance analysis - Volcano

### Info

**VOLCANO** - Underlying Volcano Comparison: HSD vs
NSD

**VOLCANO** -

### Publication Snippet

Differential expression analysis was performed using the DESeq2
package (v. 1.38.3) (Love MI, Huber W, Anders S (2014). “Moderated
estimation of fold change and dispersion for RNA-seq data withDESeq2.”
*Genome Biology*, *15*, 550. doi:10.1186/s13059-014-0550-8https://doi.org/10.1186/s13059-014-0550-8.). The
reported adjusted p-values were adjusted by . The significance level was
set to 0.05. There were a total of 1 comparison done, precisely:
HSD:NSD, from which all were visualized within the set comparison. For
each comparison, their set of entities of interest ( based on the
Significant p-values) were visualized. Note, that multiple testing
correction is done for each comparison separately.

## HEATMAP

### Info

**HEATMAP** - The heatmap was constructed based on the
following row selection: Select based on Annotation

**HEATMAP** - The rows were subsetted based on
Ensembl\_ID
:ENSMUSG00000044786,ENSMUSG00000052684,ENSMUSG00000053560,ENSMUSG00000020423,ENSMUSG00000052837,ENSMUSG00000021250,ENSMUSG00000038418,ENSMUSG00000021123,ENSMUSG00000031431,ENSMUSG00000024190

**HEATMAP** - The selection was reduced to the top
entities. Total Number: 20

**HEATMAP** - Note that the order depends on Select
based on Annotation

**HEATMAP** - The heatmap samples were colored after
Treatment

**HEATMAP** - The heatmap entities were colored after
None

**HEATMAP** - columns were clustered based on:
euclidean-distance & agglomeration method: complete

**HEATMAP** - rows were clustered based on:
euclidean-distance & agglomeration method: complete

**HEATMAP** -

### Publication Snippet

The heatmap shows all entities which Ensembl\_ID is part of the set of
ENSMUSG00000044786,ENSMUSG00000052684,ENSMUSG00000053560,ENSMUSG00000020423,ENSMUSG00000052837,ENSMUSG00000021250,ENSMUSG00000038418,ENSMUSG00000021123,ENSMUSG00000031431,ENSMUSG00000024190.
The heatmap samples were colored after Treatment. The columns were
clustered based on euclidean-distance with complete-linkage. The rows
were clustered based on euclidean-distance with complete-linkage. The
rows were scaled to visualise relative difference. The heatmap was
created using the pheatmap package(v. 1.0.12) (Kolde R (2019).
*pheatmap: Pretty Heatmaps*. R package version 1.0.12,https://CRAN.R-project.org/package=pheatmap.).

## Enrichment

### Info

**Enrichment general** The analysed gene set size:
10

**Enrichment general** Chosen Organism (needed for
translation): Mouse genes (GRCm39)

**Enrichment general** The following sets to check an
enrichment: Hallmarks,KEGG,GO\_CC

**ORA** Overrepresentation analysis was perfomed.

**ORA** The genes were taken from: LFC

**ORA** The adj. p-value threshold was set to 0.05,
whereby mutliple testing correction was : Benjamini-Hochberg

### Publication Snippet

The analysis included a gene set size of 10. When necassary the
provided IDs were translated to entrezID for , utilizing the R package
biomaRt (v. 2.54.1) (Durinck S, Spellman P, Birney E, Huber W (2009).
“Mapping identifiers for the integration of genomic datasetswith the
R/Bioconductor package biomaRt.” *Nature Protocols*, *4*,
1184-1191.Durinck S, Moreau Y, Kasprzyk A, Davis S, De Moor B, Brazma A,
Huber W (2005). “BioMart and Bioconductor: apowerful link between
biological databases and microarray data analysis.”
*Bioinformatics*, *21*, 3439-3440.). The predefined sets
to test enrichment for were: Hallmarks, KEGG, GO\_CC. Over-Representation
Analysis (ORA) was performed as implemented in the R package
clusterProfilfer (v. 4.6.2) (Wu T, Hu E, Xu S, Chen M, Guo P, Dai Z,
Feng T, Zhou L, Tang W, Zhan L, Fu x, Liu S, Bo X, Yu G
(2021).“clusterProfiler 4.0: A universal enrichment tool for
interpreting omics data.” *The Innovation*, *2*(3),100141.
doi:10.1016/j.xinn.2021.100141 https://doi.org/10.1016/j.xinn.2021.100141.Yu G, Wang L,
Han Y, He Q (2012). “clusterProfiler: an R package for comparing
biological themes among geneclusters.” *OMICS: A Journal of
Integrative Biology*, *16*(5), 284-287. doi:10.1089/omi.2011.0118https://doi.org/10.1089/omi.2011.0118.).ORA identifies
whether predefined sets of genes are overrepresented among the
differentially expressed genes. It compares the proportion of genes of
interest within the dataset to what would be expected by chance within a
so-called universe. Here the universe was chosen as the set of genes
present in the genes that were present after pre-processing. Resulting
in a total of 16125 genes. The genes were obtained from LFC. The
adjusted p-value threshold was set to 0.05, with multiple testing
correction applied using Benjamini-Hochberg.

## Enrichment results

### Hallmarks\_ENRICHMENT

- The number of found enriched terms (p.adj <0.05): 4

**Hallmarks ENRICHMENT** -

- The top 5 terms are the following (sorted by adj. p.val)

| ID | Description | GeneRatio | BgRatio | pvalue | p.adjust | qvalue | geneID | Count |
| --- | --- | --- | --- | --- | --- | --- | --- | --- |
| HALLMARK\_TNFA\_SIGNALING\_VIA\_NFKB | HALLMARK\_TNFA\_SIGNALING\_VIA\_NFKB | 8/8 | 179/3573 | 0.0000000 | 0.0000000 | 0.0000000 | 12227/14281/19252/13653/22695/16476/16477/15936 | 8 |
| HALLMARK\_HYPOXIA | HALLMARK\_HYPOXIA | 4/8 | 163/3573 | 0.0002532 | 0.0020254 | 0.0013325 | 14281/19252/22695/16476 | 4 |
| HALLMARK\_UV\_RESPONSE\_UP | HALLMARK\_UV\_RESPONSE\_UP | 3/8 | 135/3573 | 0.0025697 | 0.0137052 | 0.0090166 | 12227/14281/16477 | 3 |
| HALLMARK\_P53\_PATHWAY | HALLMARK\_P53\_PATHWAY | 3/8 | 182/3573 | 0.0060208 | 0.0240831 | 0.0158441 | 12227/14281/16476 | 3 |
| HALLMARK\_APOPTOSIS | HALLMARK\_APOPTOSIS | 2/8 | 139/3573 | 0.0360682 | 0.1039880 | 0.0684131 | 12227/16476 | 2 |

## Enrichment

### Info

**Enrichment general** The analysed gene set size:
16125

**Enrichment general** Chosen Organism (needed for
translation): Mouse genes (GRCm39)

**Enrichment general** The following sets to check an
enrichment: Hallmarks,KEGG,GO\_BP

**GSEA** Gene Set enrichment analysis was perfomed.

**GSEA** The genes were sorted by: LFC

**GSEA** Calculation based on Treatment: HSD vs. NSD

**GSEA** The adj. p-value threshold was set to 0.05,
whereby mutliple testing correction was : Benjamini-Hochberg

### Publication Snippet

The analysis included a gene set size of 16125. When necassary the
provided IDs were translated to entrezID for , utilizing the R package
biomaRt (v. 2.54.1) (Durinck S, Spellman P, Birney E, Huber W (2009).
“Mapping identifiers for the integration of genomic datasetswith the
R/Bioconductor package biomaRt.” *Nature Protocols*, *4*,
1184-1191.Durinck S, Moreau Y, Kasprzyk A, Davis S, De Moor B, Brazma A,
Huber W (2005). “BioMart and Bioconductor: apowerful link between
biological databases and microarray data analysis.”
*Bioinformatics*, *21*, 3439-3440.). The predefined sets
to test enrichment for were: Hallmarks, KEGG, GO\_BP. Gene Set Enrichment
Analysis (GSEA) was performed as implemented in the R package
clusterProfilfer (v. 4.6.2) (Wu T, Hu E, Xu S, Chen M, Guo P, Dai Z,
Feng T, Zhou L, Tang W, Zhan L, Fu x, Liu S, Bo X, Yu G
(2021).“clusterProfiler 4.0: A universal enrichment tool for
interpreting omics data.” *The Innovation*, *2*(3),100141.
doi:10.1016/j.xinn.2021.100141 https://doi.org/10.1016/j.xinn.2021.100141.Yu G, Wang L,
Han Y, He Q (2012). “clusterProfiler: an R package for comparing
biological themes among geneclusters.” *OMICS: A Journal of
Integrative Biology*, *16*(5), 284-287. doi:10.1089/omi.2011.0118https://doi.org/10.1089/omi.2011.0118.). GSEA evaluates
whether predefined sets of genes show statistically significant
differences in expression between two biological states. It considers
the entire ranked list of genes, thus providing insights into pathways
that might be enriched even if individual genes do not reach
significance. The genes were sorted by LFC, whereby the calculation was
done for Treatment for HSD vs. NSD. The adjusted p-value threshold was
set to 0.05, with multiple testing correction applied using
Benjamini-Hochberg.

## Enrichment results

### Hallmarks\_ENRICHMENT

- The number of found enriched terms (p.adj <0.05): 6

**Hallmarks ENRICHMENT** -

- The top 5 terms are the following (sorted by adj. p.val)

| ID | Description | setSize | enrichmentScore | NES | pvalue | p.adjust | qvalue | rank | leading\_edge | core\_enrichment |
| --- | --- | --- | --- | --- | --- | --- | --- | --- | --- | --- |
| HALLMARK\_TNFA\_SIGNALING\_VIA\_NFKB | HALLMARK\_TNFA\_SIGNALING\_VIA\_NFKB | 178 | 0.5550914 | 2.633560 | 0.0000000 | 0.0000000 | 0.0000000 | 1253 | tags=26%, list=8%, signal=24% | 19252/14282/13653/14281/18626/16476/15370/22695/17691/12227/12608/15936/16477/11852/16176/16598/211770/19225/20620/16193/230738/13654/21664/17872/56706/20310/18035/227659/15205/12515/50723/18578/21815/23872/16197/16160/23849/230734/16601/17118/17210/54446/12044/21930/12522/20971 |
| HALLMARK\_OXIDATIVE\_PHOSPHORYLATION | HALLMARK\_OXIDATIVE\_PHOSPHORYLATION | 197 | -0.3660331 | -1.791360 | 0.0000064 | 0.0001596 | 0.0001311 | 3687 | tags=36%, list=23%, signal=28% | 18597/12369/64655/66052/30055/16922/11973/51798/68194/67834/16828/13063/73834/12859/17448/66091/30059/67264/11740/225887/22272/66152/71679/12866/68198/12034/57423/12861/30057/11974/11957/67126/69833/269951/54405/11958/18105/228033/11946/14297/72900/110323/11950/66495/17992/66046/15526/28185/28080/66377/11739/66290/214952/231086/110446/15926/12868/17713/66335/12856/66142/407785/66525/67530/69772/109672/68375/56451/17993/11655/67942 |
| HALLMARK\_MTORC1\_SIGNALING | HALLMARK\_MTORC1\_SIGNALING | 196 | -0.3607102 | -1.764161 | 0.0000232 | 0.0003872 | 0.0003179 | 3905 | tags=41%, list=24%, signal=32% | 17768/107272/18107/67895/56088/18655/56418/16993/12317/17252/20775/66249/53333/107513/21753/16828/20135/74117/15277/16414/73834/13361/56480/14884/19324/15357/11938/104112/67890/74205/56305/74185/23996/68275/15528/26941/20878/21991/22256/208715/192193/68801/68278/13595/70699/72157/20893/15452/14385/59029/78925/13806/14433/665563/15526/103963/93692/54353/22433/56529/11639/12450/22027/235293/16011/15926/12330/20491/27407/667034/14381/64136/26432/27966/74754/112407/107476/18817/20525/20867/18451 |
| HALLMARK\_MYC\_TARGETS\_V1 | HALLMARK\_MYC\_TARGETS\_V1 | 197 | -0.3381885 | -1.655089 | 0.0000990 | 0.0012379 | 0.0010164 | 4465 | tags=38%, list=28%, signal=28% | 53607/67204/14208/20588/12462/18148/12464/231872/20382/13690/70247/20174/18655/67097/22630/78655/381760/57296/16828/17220/14113/13204/433702/105148/12566/27041/26445/12261/19385/110074/11777/230908/26440/103573/20383/23996/15528/19988/12034/74326/26446/16898/18972/233870/17218/108062/18263/20639/23983/15452/59029/11792/19166/22327/28185/12428/99138/11431/19384/12237/22195/19826/56150/106344/12330/50995/68092/22627/27966/20641/110639/85305/68011/56351/22171 |
| HALLMARK\_COAGULATION | HALLMARK\_COAGULATION | 87 | -0.4145408 | -1.788772 | 0.0003339 | 0.0033390 | 0.0027415 | 3089 | tags=37%, list=19%, signal=30% | 12759/17385/227753/14058/17395/11502/54368/18787/223864/16416/229445/14723/11812/12334/22388/16784/16952/76453/108078/12258/11843/19128/18441/12371/76467/20196/21859/18792/67603/14066/17390/56744 |

### KEGG\_ENRICHMENT

- The number of found enriched terms (p.adj <0.05): 4

**KEGG ENRICHMENT** -

- The top 5 terms are the following (sorted by adj. p.val)

| ID | Description | setSize | enrichmentScore | NES | pvalue | p.adjust | qvalue | rank | leading\_edge | core\_enrichment |
| --- | --- | --- | --- | --- | --- | --- | --- | --- | --- | --- |
| KEGG\_PARKINSONS\_DISEASE | KEGG\_PARKINSONS\_DISEASE | 108 | -0.4935969 | -2.253620 | 0.0000000 | 0.0000024 | 0.0000023 | 4959 | tags=53%, list=31%, signal=37% | 17722/333182/22273/11949/11951/67680/230075/67130/17708/66594/67273/67003/66108/66052/68194/13063/12859/66091/67264/11740/225887/78330/22272/66152/71679/12866/68198/20617/12861/11957/67126/54405/228033/11946/72900/110323/11950/66495/17992/66046/12367/28080/66377/11739/12862/22195/12868/56791/66142/66218/407785/67738/67530/68375/12371/17993/67942 |
| KEGG\_OXIDATIVE\_PHOSPHORYLATION | KEGG\_OXIDATIVE\_PHOSPHORYLATION | 109 | -0.4895433 | -2.240331 | 0.0000001 | 0.0000061 | 0.0000057 | 5437 | tags=62%, list=34%, signal=42% | 12858/12864/68197/66916/11966/76429/66237/17722/333182/22273/11949/11951/67680/230075/67130/17708/66594/67273/67003/66144/66108/67895/11972/66052/11973/68194/73834/12859/66091/67264/225887/78330/22272/66152/71679/12866/68198/57423/12861/11974/11957/67126/69875/54405/11958/228033/11946/72900/110323/11950/66495/17992/66046/28080/66377/11964/12862/66290/12868/66335/12856/66142/66218/407785/67530/68375/17993/67942 |
| KEGG\_ALZHEIMERS\_DISEASE | KEGG\_ALZHEIMERS\_DISEASE | 143 | -0.4065408 | -1.925576 | 0.0000021 | 0.0000931 | 0.0000880 | 4738 | tags=52%, list=29%, signal=37% | 19164/12314/11949/11951/67680/230075/67130/12015/15925/17708/66594/18795/67273/67003/19056/66108/16956/12369/19058/66052/68194/78943/13063/12859/11785/66091/67264/11938/225887/78330/12370/22272/66152/71679/12313/12866/68198/20617/14811/12861/12315/11957/12568/67126/59287/54405/228033/11946/72900/110323/14433/12334/11950/66495/17992/66046/12367/14812/12122/28080/18798/20192/66377/12862/11820/12868/66142/66218/407785/54652/67530/68375/12371/17993/67942 |
| KEGG\_HUNTINGTONS\_DISEASE | KEGG\_HUNTINGTONS\_DISEASE | 154 | -0.4001709 | -1.914644 | 0.0000021 | 0.0000931 | 0.0000880 | 4988 | tags=51%, list=31%, signal=35% | 15194/69654/333182/237336/22273/11949/11773/11951/67680/230075/67130/17708/66594/18795/67273/67003/217864/66108/13191/66052/68194/13385/20466/13063/12859/20021/74325/66091/67264/21780/11740/327954/54152/225887/78330/12370/22272/66152/71679/12866/68198/12064/12861/69241/11957/67126/69833/54405/105000/228033/11946/72900/110323/11950/66495/17992/26427/66046/12367/14812/28080/18798/208647/66377/11739/12862/67710/12868/66142/66218/407785/67738/67530/68375/12371/17993/67942/21817 |
| KEGG\_PROTEIN\_EXPORT | KEGG\_PROTEIN\_EXPORT | 22 | -0.5704451 | -1.822860 | 0.0030073 | 0.1058553 | 0.1000307 | 2880 | tags=45%, list=18%, signal=37% | 20813/67398/69019/66384/56529/66212/53421/66541/66624/20335 |

### GO\_BP\_ENRICHMENT

- The number of found enriched terms (p.adj <0.05): 24

**GO\_BP ENRICHMENT** -

- The top 5 terms are the following (sorted by adj. p.val)

| ID | Description | setSize | enrichmentScore | NES | pvalue | p.adjust | qvalue | rank | leading\_edge | core\_enrichment |
| --- | --- | --- | --- | --- | --- | --- | --- | --- | --- | --- |
| GOBP\_POSITIVE\_REGULATION\_OF\_ACUTE\_INFLAMMATORY\_RESPONSE | GOBP\_POSITIVE\_REGULATION\_OF\_ACUTE\_INFLAMMATORY\_RESPONSE | 19 | 0.7484267 | 2.234386 | 1.45e-05 | 0.0153045 | 0.014562 | 251 | tags=26%, list=2%, signal=26% | 18413/16176/19225/16193/11501 |
| GOBP\_SKELETAL\_MUSCLE\_CELL\_DIFFERENTIATION | GOBP\_SKELETAL\_MUSCLE\_CELL\_DIFFERENTIATION | 43 | 0.6058505 | 2.230243 | 7.60e-06 | 0.0153045 | 0.014562 | 1575 | tags=26%, list=10%, signal=23% | 13653/14281/15370/12227/13654/17260/17261/13813/77578/13207/224640 |
| GOBP\_PROTON\_TRANSMEMBRANE\_TRANSPORT | GOBP\_PROTON\_TRANSMEMBRANE\_TRANSPORT | 107 | -0.4253468 | -1.899171 | 1.71e-05 | 0.0153045 | 0.014562 | 3291 | tags=40%, list=20%, signal=32% | 11973/68073/68055/12859/57738/66114/11740/68020/66152/71679/26941/212933/12034/57423/12861/56632/11974/11957/236794/67126/83429/11958/228033/11946/331004/110323/105675/11950/269356/17992/57816/28080/11964/11739/12862/66290/12868/66335/12856/66142/109672/67942/22229 |
| GOBP\_NUCLEOSIDE\_PHOSPHATE\_BIOSYNTHETIC\_PROCESS | GOBP\_NUCLEOSIDE\_PHOSPHATE\_BIOSYNTHETIC\_PROCESS | 197 | -0.3555194 | -1.753945 | 1.07e-05 | 0.0153045 | 0.014562 | 3328 | tags=35%, list=21%, signal=28% | 30963/14913/68073/20135/68055/71743/66114/269614/237823/67054/54391/110074/11637/104112/353172/74205/11534/71679/71562/20617/11566/68870/192185/57423/56632/106564/11957/67126/68801/22169/73836/11958/228033/11946/15452/74559/13806/665563/11950/108147/19063/28080/80914/11964/70456/56348/69225/11639/11821/67993/70789/110446/319945/11541/75456/236900/667034/171567/266645/110639/107476/85305/223646/15930/54195/22171/67942/22271/79059 |
| GOBP\_CHROMATIN\_ORGANIZATION | GOBP\_CHROMATIN\_ORGANIZATION | 488 | 0.3046313 | 1.600807 | 4.80e-06 | 0.0153045 | 0.014562 | 3731 | tags=31%, list=23%, signal=24% | 16598/224836/360198/100683/216848/50708/252838/228790/108829/116848/407823/320790/22589/192285/277250/53892/97908/214162/231051/214899/18602/15184/218850/622675/73251/381022/20230/244059/328572/104248/67772/72895/68094/21652/20926/108155/212712/71458/14055/192195/67155/110958/53890/20185/68142/18193/235134/103554/75751/94246/224826/15081/233532/494448/57261/107976/235626/56335/107932/207165/238247/320538/67246/57749/20591/233490/66867/69188/14462/232811/20613/268564/22289/170787/69386/17257/216850/231386/75410/12005/233545/114642/66505/234135/75560/233875/217578/19651/270058/242466/68968/223828/52609/110147/74016/13018/93760/17954/229675/53325/17345/17450/320795/193796/208043/101612/225876/320713/237339/224903/244349/54343/75605/20664/230936/69612/233900/76719/20918/227867/52808/70645/12418/73247/104263/73884/17192/15260/225888/105787/68845/59035/21415/19820/71330/71389/214133/68703/12648/81601/14534/109275/170644/320376/16969/19650/20184/319156/671535 |
